# Security Analysis and Improvements of Two-Factor Mutual Authentication with Key Agreement in Wireless Sensor Networks

**DOI:** 10.3390/s140406443

**Published:** 2014-04-09

**Authors:** Jiye Kim, Donghoon Lee, Woongryul Jeon, Youngsook Lee, Dongho Won

**Affiliations:** 1 College of Information and Communication Engineering, Sungkyunkwan University, 2066 Seobu-Ro, Jangan-Gu, Suwon-Si, Gyeonggi-Do 440-746, Korea; E-Mails: jykim@security.re.kr (J.K.); dhlee@security.re.kr (D.L.); 2 Department of Cyber Security and Police, Gwangju University, 277 Hyodeok-Ro, Nam-Gu, Gwangju-Si 503-703, Korea; E-Mail: wrjeon@gwangju.ac.kr; 3 Department of Cyber Investigation Police, Howon University, 64 Howon University 3Gil, Impi-Myeon, Gunsan-Si, Jeonrabuk-Do 573-718, Korea; E-Mail: ysooklee@howon.ac.kr; 4 College of Information and Communication Engineering, Sungkyunkwan University, 2066 Seobu-Ro, Jangan-Gu, Suwon-Si, Gyeonggi-Do 440-746, Korea

**Keywords:** wireless sensor networks (WSNs), mutual authentication, key agreement, two-factor authentication, passwords, smart cards

## Abstract

User authentication and key management are two important security issues in WSNs (Wireless Sensor Networks). In WSNs, for some applications, the user needs to obtain real-time data directly from sensors and several user authentication schemes have been recently proposed for this case. We found that a two-factor mutual authentication scheme with key agreement in WSNs is vulnerable to gateway node bypassing attacks and user impersonation attacks using secret data stored in sensor nodes or an attacker's own smart card. In this paper, we propose an improved scheme to overcome these security weaknesses by storing secret data in unique ciphertext form in each node. In addition, our proposed scheme should provide not only security, but also efficiency since sensors in a WSN operate with resource constraints such as limited power, computation, and storage space. Therefore, we also analyze the performance of the proposed scheme by comparing its computation and communication costs with those of other schemes.

## 1. Introduction

A wireless sensor network (WSN) is composed of a number of sensors (tens to thousands) that are deployed to collect data in a target area [1,2]. The number of potential applications for WSNs is increasing in various fields, including environmental monitoring, healthcare, agriculture, manufacturing, military sensing and tracking, and disaster alert [1–5]. The design of a specific WSN is dependent on the given application and the environment under which it operates [1]. In addition, sensors in a WSN operate with resource constraints such as limited power, computation, and storage space [1,3,6–8]. In WSNs, user queries are generally transmitted to the gateway [1,3,8,9]. However, for some applications, the user needs to obtain real-time data directly from sensors [1,3,8,9]. In this case, only legitimate users should be able to access the WSN.

Several schemes for user authentication in WSNs have been proposed recently. Wong *et al.* [10] proposed a user authentication scheme that uses only one-way hash functions for computation efficiency on sensor nodes [10]. However, Das [3] pointed out that Wong *et al.*'s scheme does not prevent many logged-in users with the same login-ID threats and stolen-verifier attacks [3]. Das [3] proposed a two-factor user authentication in WSNs using a smart card and a password instead of maintaining a password/verifier table [3]. Other researchers, however, pointed out that Das' scheme still has security flaws. Chen and Shih [11] insisted that Das' scheme does not provide mutual authentication, and proposed a mutual authentication scheme between the user, the gateway, and the sensor node [11]; He *et al.* [9] said that Das' scheme has security weaknesses against insider attacks and impersonation attacks [9]; and Khan and Alghathbar [4] pointed out that Das' scheme is vulnerable to gateway node bypassing attacks and privileged-insider attacks [4]. In 2012, Vaidya *et al.* [12] pointed out that the schemes proposed by Das [3], Kan and Alghathbar [4] and Chen and Shih [11] are all insecure against stolen smart card attacks and sensor node impersonation attacks with node capture attacks and do not provide key agreement [12]. Therefore, they proposed a novel two-factor mutual authentication and key agreement scheme to prevent these attacks. In addition, they insisted that computational costs for gateway and sensor nodes in their proposed scheme are not so high. However, we found that their proposed scheme still has security flaws.

In this paper, we present that gateway node bypassing attacks and user impersonation attacks are possible using secret data stored in a sensor or an attacker's own smart card in Vaidya *et al.*'s scheme. Additionally, we propose an improved scheme that eliminates such security weaknesses from Vaidya *et al.*'s scheme. We verify that the proposed scheme is secure against possible attacks. We also analyze the performance of the proposed scheme by comparing its computation cost and communication cost with those of other schemes.

The remainder of the paper is organized as follows. Section 2 presents a review of Vaidya *et al.*'s scheme. Section 3 is devoted to analyzing the security of Vaidya *et al.*'s scheme. Section 4 proposes the improved scheme. Section 5 analyzes the security of the proposed scheme against possible attacks. Section 6 is devoted to analyzing the performance of the proposed scheme and Section 7 concludes this paper.

## 2. Review of Vaidya *et al.*'s Scheme

There are three communication parties in Vaidya *et al.*'s scheme [12]: a user, a gateway node, and a sensor node. This scheme is composed of four phases: registration phase, login phase, authentication-key agreement phase, and password change phase. We describe each phase in detail in Sections 2.1–2.4, and Table 1 shows the notations used in the remainder of the paper.

Registration phase begins when the user sends a registration request with his/her identity and a hashed password to the gateway node. Then, the gateway node personalizes a smart card for the user and sends it to him/her as a response to the registration request. In the registration phase, all these communication messages are transmitted in secure channels.

Login phase begins when the user inserts his/her smart card into the terminal and inputs his/her identity and password. After the verification of the user's input value, the smart card computes and sends the authentication request to the gateway node. When the gateway node receives the authentication request from the user side, the authentication-key agreement phase begins. The gateway node verifies whether the authentication request comes from a legitimate user. If the verification is successful, the gateway node sends the authentication request to a sensor node which can respond to a request or a query from the user. In this phase, three authentication requests are transmitted. The first request is from the gateway node to the sensor node, the second is from the sensor node to the gateway node, and the final is from the gateway node to the user. As stated, when one party receives an authentication request, the party verifies its validity and sends a new authentication request to the other party. In login phase and authentication-key agreement phase, these request messages are transmitted in insecure channels. If all verifications are passed successfully, the user and the sensor node then share the session key for communication. The password change phase begins whenever the user wants to change his/her password. In the password change phase, the user side does not have to communicate with other parties.

### 2.1. Registration Phase

**Figure 1.**
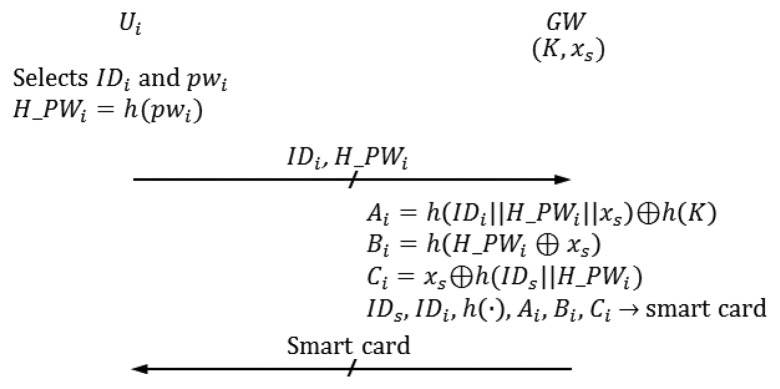
Registration phase of Vaidya *et al.*'s scheme [12].

We describe the registration phase in this subsection. *U_i_* selects *ID_i_* and *pw_i_*, computes *H*_*PW_i_*=*h*(*pw_i_*) and sends the registration request {*ID_i_,h*(*pw_i_*)} to *GW*. Then, *GW* personalizes a smart card for *U_i_* and sends it to *U_i_*. Figure 1 shows the registration phase of Vaidya *et al.*'s scheme.


R-1*U_i_* selects *ID_i_* and.*PW_i_*R-2*U_i_* computes *H*_*PW_i_*=*h*(*pw_i_*)*U_i_* sends a registration request {*ID_i_*, *H*_*PW_i_*} to *GW* in secure channels (it was not mentioned whether the registration request from *U_i_* to *GW* is sent by secure channels [12], but we guess that it is sent this way).R-3*GW* computes the following when it receives the registration request from *U_i_*. *A_i_*=*h*(*ID_i_*∥*H_PW_i_*∥*x_s_*)⊕*h*(*K*) *B_i_*=*h*(*H_PW_i_*⊕*x_s_*) *C_i_*=*x_s_*⊕*h*(*ID_s_*∥*H_PW_i_*)*GW* personalizes the smart card with *ID_s_*, *ID_i_*, *h*(·), *A_i_*, *B_i_* and *C_i_*.*GW* sends the smart card to *U_i_* in secure channels.


Meanwhile, *SID_j_* and a secret value *x_s_* generated by *GW* are stored in *S_j_* before it is deployed into a target field.

### 2.2. Login Phase

**Figure 2.**
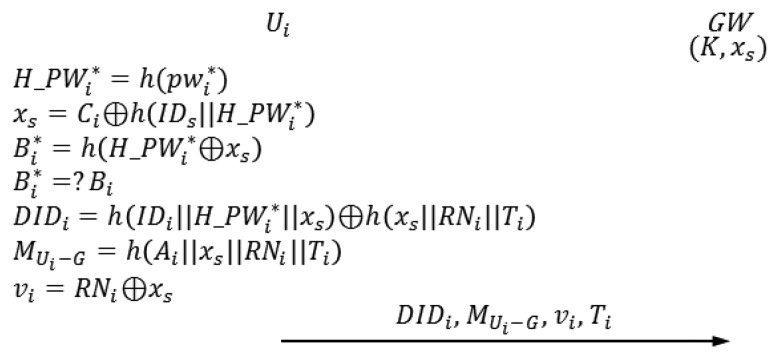
Login phase of Vaidya *et al.*'s scheme [12].

The login phase begins when *U_i_* inserts *U_i_*'s smart card into a terminal and inputs $ID_{i}^{*}$ and $pw_{i}^{*}$. In this phase, *U_i_* sends the authentication request to *GW*. Figure 2 illustrates the login phase of Vaidya *et al.*'s scheme.


L-1*U_i_* inserts *U_i_*'s smart card into a terminal and inputs 
$ID_{i}^{*}$ and 
$pw_{i}^{*}$L-2The smart card computes the following.
$H\_PW_{i}^{*}=h\left(pw_{i}^{*}\right)$
$x_{s}=C_{i}⊕h(ID_{s}\|H\_PW_{i}^{*})$
$B_{i}^{*}=h(H\_PW_{i}^{*}⊕x_{s})$The smart card compares 
$B_{i}^{*}$ with *B_i_*. If, 
$B_{i}^{*}=B_{i}$, then the next step proceeds; otherwise, this phase is aborted.L-3The smart card generates a random nonce *R_Ni_* and computes the following. *T_i_* is the current timestamp of *U_i_* system. 
$DID_{i}=h(ID_{i}\|H\_PW_{i}^{*}\|x_{s})h(x_{s}\|RN_{i}\|T_{i})$ *M_Ui_*_−_*_G_*=*h*(*A_i_*∥*x_s_*∥*R_Ni_*∥*T_i_*) *v_i_*=*R_Ni_*⊕*x_s_*The smart card sends the authentication request {*DID_i_*, *M_Ui_*_−_*_G_*, *v_i_*, *T_i_*} to *GW*.


### 2.3. Authentication-Key Agreement Phase

**Figure 3.**
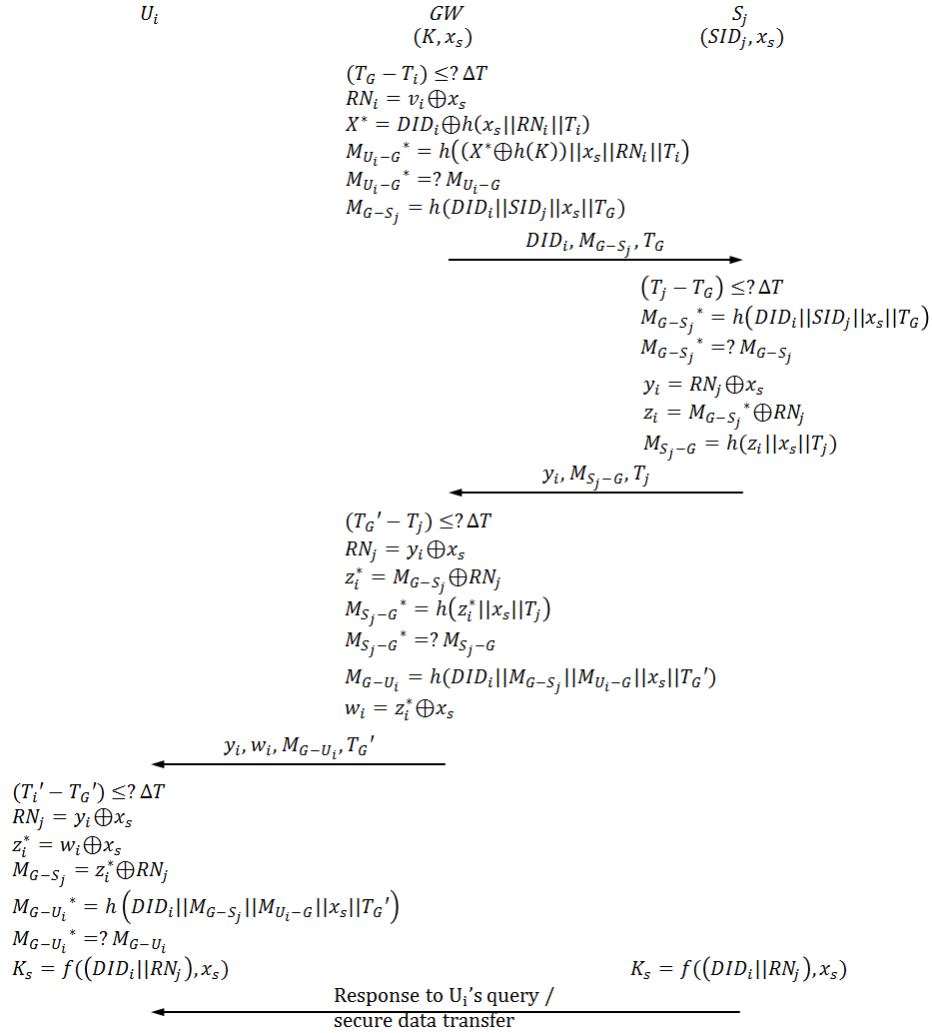
Authentication-key agreement phase of Vaidya *et al.*'s scheme [12].

When *GW* receives the authentication request from *U_i_*, the authentication-key agreement phase begins. In this phase, *U_i_*, *GW*, *S_j_* and send and receive authentication requests from one another. Figure 3 depicts the authentication-key agreement phase of Vaidya *et al.*'s scheme. The following describes this process in detail.


A-1*GW* checks if (*T_G_*−*T_i_*) ≤ Δ*T*, where *T_G_* is the current timestamp of *GW* system, and Δ*T* is the maximum permitted transmission delay time. If (*T_G_*−*T_i_*) ≤ Δ*T*, then the next step proceeds; otherwise, this phase is aborted.A-2*GW* computes the following. *R_Ni_*=*v_i_*⊕*x_s_* X*=*DID_i_*⊕*h*(*x_s_*∥*R_Ni_*∥*T_i_*) *M_Ui_*_−__G_*=*h*((X*⊕*h*(*K*))∥*x_s_*∥*R_Ni_*∥*T_i_*)*GW* compares *M_Ui_*_−__G_* with *M_Ui_*_−__G_*. If *M_Ui_*_−__G_* =*M_Ui_*_−_*_G_*, then the next step proceeds; otherwise, this phase is aborted.A-3*GW* computes *M_G_*_−_*_Sj_*=*h*(*DID_i_*∥*SID_j_*∥*x_s_*∥*T_G_*). *T_G_* is the current timestamp of *GW* system. *S_j_* is the nearest sensor node that can respond to *U_i_*'s request.*GW* sends the authentication request {*DID_i_*, *M_G_*_−_*_Sj_*, *T_G_*} to *S_j_*.A-4*GW* checks if (*T_j_* − *T_G_*) ≤ Δ*T*, where *T_j_* is the current timestamp of *S_j_* system.If (*T_j_* −*T_G_*) ≤ Δ*T*, then the next step proceeds; otherwise, this phase is aborted.A-5*S_j_* computes *M_G_*_−__Sj_*=*h*(*DID_i_*∥*SID_j_*∥*x_s_*∥*T_G_*).*S_j_* compares *M_G_*_−__Sj_* with *M_G_*_−_*_Sj_*. If *M_G_*_−__Sj_* = *M_G_*_−_*_Sj_*, then the next step proceeds; otherwise, this phase is aborted.A-6*S_j_* generates a random nonce *RN_j_* and computes the following. *y_i_* = *RN_j_*⊕*x_s_* *z_i_* = *M_G_*_−__Sj_*⊕*RN_j_* *M_Sj_*_−_*_G_* = *h*(*z_i_*∥*x_s_*∥*T_j_*)*S_j_* sends the authentication request {*y_i_*, *M_Sj_*_−_*_G_*, *T_j_*} to *GW*.A-7*GW* checks if (*T_G_*′ − *T_j_*) ≤ Δ*T*, where *T_G_*′ is the current timestamp of *GW* system.If (*T_G_*′−*T_j_*) ≤ Δ*T*, then the next step proceeds; otherwise, this phase is aborted.A-8*GW* computes the following. *RN_j_*=*y_i_* ⊕ *x_s_* 
$z_{i}^{*}=M_{G-S_{j}}⊕RN_{j}$ 
$M_{S_{j}-G}*=h\left(z_{i}^{*}\|x_{s}\|T_{j}\right)$*GW* compares *M_Sj_*_−__G_* with *M_Sj_*_−_*_G_*. If *M_Sj_*_−__G_* = *M_Sj_*_−_*_G_*, then the next step proceeds; otherwise, this phase is aborted.A-9*GW* computes the following. *M_G_*_−_*_Ui_* = *h*(*DID_i_*∥*M_G_*_−_*_Sj_*∥*M_Ui_*_−_*_G_*∥*x_s_*∥*T_G_*′) 
$w_{i}=z_{i}^{*}x_{s}$*GW* sends the authentication request {*y_i_*, *w_i_*, *M_G_*_−_*_Ui_*, *T_G_*′} to *U_i_*.A-10*U_i_* checks if (*T_i_*′ − *T_G_*′) ≤ Δ*T*, where *T_i_*′ is the current timestamp of *U_i_* system. If (*T_i_*′ − *T_G_*′) ≤ Δ*T*, then the next step proceeds; otherwise, this phase is aborted.A-11The smart card computes the following. *RN_j_* = *y_i_* ⊕ *x_s_* 
$z_{i}^{*}=w_{i}⊕x_{s}$ 
$M_{G-S_{j}}=z_{i}^{*}⊕RN_{j}$ *M_G_*_−__Ui_* = *h*(*DID_i_*∥*M_G_*_−_*_Sj_*∥*M_Ui_*_−_*_G_*∥*x_s_*∥*T_G_*′)The smart card compares *M_G_*_−__Ui_* with *M_G_*_−_*_Ui_*. If *M_G_*_−__Ui_* = *M_G_*_−_*_Ui_*, then mutual authentication between *U_i_* and *S_j_* is completed successfully; otherwise, this phase is aborted.A-12The smart card computes *K_s_* = *f*((*DID_i_*∥*R_Ni_*),*x_s_*) to obtain a session key for commu*_ni_*cation with *S_j_*. Meanwhile, *S_j_* also computes *K_S_* = *f*((*DID_i_*∥*R_Ni_*),*x_s_*) to share a session key with *U_i_*.


### 2.4. Password Change Phase

The password change phase proceeds when *U_i_* changes *U_i_*'s existing password to a new one. In the password change phase, *U_i_* does not communicate with *GW*.


P-1*U_i_* inserts *U_i_*'s smart card into a terminal and inputs, 
$ID_{i}^{*}$, 
$pw_{i}^{*}$, and *pw_ni_*. *pw_ni_* is *U_i_*'s new password.P-2The smart card computes the following. 
$H\_PW_{i}^{*}=h\left(pw_{i}^{*}\right)$ 
$x_{s}=C_{i}⊕h(ID_{s}\|H\_PW_{i}^{*})$ 
$B_{i}^{*}=h(H\_PW_{i}^{*}⊕x_{s})$The smart card compares 
$B_{i}^{*}$ with *B_i_*. If 
$B_{i}^{*}=B_{i}$, then the next step proceeds; otherwise, this phase is aborted.P-3The smart card computes the following. 
$H\_PW_{ni}=h\left(pw_{ni}\|RN_{r}^{*}\right)$ 
$A_{ni}=A_{i}⊕h(ID_{i}\|H\_PW_{i}^{*}\|x_{s})⊕h\left(ID_{i}\|H\_PW_{ni}\|x_{s}\right)$ *B_ni_*=*h*(*H_PW_ni_*⊕*x_s_*) *C_ni_*=*x_s_*⊕*h*(*ID_s_*∥*H_PW_ni_*)The smart card replaces the existing values *A_i_*, *B_i_*, and *C_i_* with the new values *A_ni_*, *B_ni_*, and *C_ni_*.


## 3. Security Analysis of Vaidya *et al.*'s Scheme

In this section, we analyze the security of Vaidya *et al.*'s scheme. We found that gateway node bypassing attacks are possible in Vaidya *et al.*'s scheme if an attacker captures a sensor node and extracts secret values stored in it. Additionally, an attacker can know secret values *x_s_* and *h*(*K*) from the attacker's own smart card and use them for user impersonation attacks or gateway node bypassing attacks.

In Sections 3.1–3.3, we describe possible attacks in Vaidya *et al.*'s scheme in detail. We assume that an attacker can eavesdrop on or intercept all messages sent or received between communication parties. We also assume that an attacker can read data stored in a smart card in any manner like in the related works [2,6,13–16]. In addition, we have to note that data stored in sensor nodes are not secure since an attacker can capture sensor nodes that are deployed in unattended environments and can then extract data from them.

### 3.1. Gateway Node Bypassing Attacks Using Secret Data Stored in a Sensor Node

In Vaidya *et al.*'s scheme, if an attacker extracts the secret data *x_s_* from a sensor node, he/she can impersonate *GW* and communicate with *U_i_*. These attacks proceed as explained below. *U_α_* denotes an attacker here.


Step 1*U_α_* extracts *x_s_* and *SID_j_* from a sensor node captured in the WSN.Step 2Login phase begins when *U_i_* wants to access to the WSN as in Section 2.2. When *U_i_* sends the authentication request {*DID_i_*, *M_Ui_*_−_*_G_*, *v_i_*, *T_i_*} to *GW*, *U_α_* eavesdrops on it.Step 3*U_α_* computes the following using *x_s_*, *SID_j_* and {*DID_i_*, *M_Ui_*_−_*_G_*, *v_i_*, *T_i_*}. *T_α_* and *T_α_*′ denote the current timestamp of *U_α_* system, and *T_α_* < *T_α_*′. *U_α_* generates a random nonce *RN_α_*. *y_i_*=*RN_α_*⊕*x_s_* *M_G-Sj_* = *h*(*DID_i_*∥*SID_j_* ∥*x_s_* ∥*T_α_*) 
$z_{i}^{*}=M_{G-S_{j}}⊕RN_{\alpha }$ 
$w_{i}=z_{i}^{*}⊕x_{s}$ *M_G_*_−__Ui_*=*h*(*DID_i_*∥*M_G_*_−_*_Sj_*∥*M_Ui_*_−_*_G_*∥*x_s_*∥*T_α_*′)*U_α_* forges the authentication request sent from *GW* to *U_i_* in authentication-key agreement phase using {*y_i_*, *w_i_*, *M_G_*_−_*_Ui_*),*T_α_*′}.Step 4When *U_i_* receives {*y_i_*, *w_i_*, *M_G_*_−_*_Ui_*, *T_α_*′} from *U_α_*, *U_i_* checks if (*T_U_*′−*T_α_*′) ≤ Δ*T*, where (*T_U_*′ is the current timestamp of *U_i_* system. If (*T_U_*′−*T_α_*′) ≤ Δ*T*, then the next step proceeds; otherwise, this phase is aborted.Step 5The smart card computes the following. *RN_α_* = *y_i_* ⊕ *x_s_* 
$z_{i}^{*}=w_{i}⊕x_{S}$ 
$M_{G-U_{i}}=h(DID_{i}\|M_{G-S_{j}}\|M_{U_{i}-G}\|x_{s}\|T_{\alpha }\prime )$ *M_G_*_−__Ui_*=*h*(*DID_i_*∥*M_G_*_−_*_Sj_*∥*M_Ui_*_−_*_G_*∥*x_s_*∥*T_α_*′)The smart card compares *M_G_*_−_*_Ui_*with *M_G_*_−__Ui_*. Since *M_G_*_−_*_Ui_* = *M_G_*_−__Ui_*, *U_i_* regards {*y_i_*, *w_i_*, *M_G_*_−_*_Ui_*,*Tα′*} as being transmitted from *GW*. Therefore, *U_α_* can communicate with *U_i_* using the session key *K_s_* = *f*((*DID_i_*∥*RN_α_*), *x_s_*).


### 3.2. User Impersonation Attacks Using an Attacker's Own Smart Card

If an attacker *U_α_* registers with GW, *U_α_* receives the smart card personalized with *U_α_*'s own identity and password, *ID_α_* and *pw_α_*. *U_α_* can compute *x_s_* and *h*(*K*) using *ID_α_*, *pw_α_*, and secret values stored in the smart card.


Step 1As shown in the Section 2.1, *U_α_* selects *ID_α_* and *pw_α_*.Step 2*U_α_* computes *H*_*PW_α_*=*h*(*pw_α_*).*U_α_* sends the registration request {*ID_α_*, *h*(*pw_α_*)} to *GW*.Step 3*GW* computes the following when it receives the registration request from *U_α_*. *A_α_* = *h*(*ID_α_*∥*H*_*PW_α_*∥*x_s_*)⊕*h*(*K*) *B_α_*=*h*(*H*_*PW_α_*⊕*x_s_*) *C_α_*=*x_s_* ⊕ *h*(*ID_s_*∥*H*_*PW_α_*)*GW* personalizes the smart card with *ID_s_*, *ID_α_*, *h*(·), *A_α_*, *B_α_* and *C_α_*.*GW* sends the smart card to *U_α_*.Step 4*U_α_* reads *ID_s_*, *ID_α_*, *A_α_*, *B_α_*, and *C_α_* from the smart card.*U_α_* can know *x_s_* and *h*(*K*) by computing the following. *x_s_*= *C_α_* ⊕ *h*(*ID_s_*∥*H*_*PW_α_*) *h*(*K*) = *A_α_* ⊕ *h*(*ID_α_*∥*H*_*PW_α_*∥ *x_s_*)


*U_α_* can impersonate a legitimate user who has registered with *GW* using *x_s_* and *h*(*K*). In addition, *U_α_* can also log in with any temporary identity that does not actually exist.

#### 3.2.1. Logging in with Any Temporary Identity

We describe the process where *U_α_* logs in with any temporary identity that does not actually exist using *x_s_* and *h*(*K*).


Step 1*U_α_* selects any temporary identity and password *ID_β_* and *pw_β_*. *U_α_* computes the authentication request as follows. *T_α_* denotes the current timestamp of *U_α_* system, and *RN_α_* is a random nonce generated by *U_α_*. 
$H_{P}W_{\beta }^{*}=h(pw_{\beta })$ 
$A_{\beta }=h(ID_{\beta }\|H\_W_{\beta }^{*}\|x_{s})⊕h(K)$ 
$DID_{\beta }=h(ID_{\beta }\|H\_PW_{\beta }^{*}||x_{s})⊕h(x_{s}\|RN_{\alpha }\|T_{\alpha })$ *M_Uβ_*_−_*_G_*=*h*(*A_β_*∥*x_s_*∥*RN_α_*∥*T_α_*) *v_β_*=*RN_α_* ⊕ *x_s_**U_α_* sends the authentication request {*DID_β_*, M*_Uβ_*_−_*_G_*, *v_β_*, *T_α_*} to *GW*.Step 2When *GW* receives the authentication request, *GW* checks if (*T_G_*−*T_α_*) ≤ Δ*T*, where *T_G_* is the current timestamp of *GW* system. If (*T_G_*−*T_α_*) ≤ Δ*T*, then the next step proceeds; otherwise, this phase is aborted.Step 3*GW* computes the following. *RN_α_*=*v_β_*⊕ *x_s_* X*=*DID_β_* ⊕ *h*(*x_s_*∥*RN_α_*∥*T_α_*) *M_Uβ_*_−__G_* = *h*((X* ⊕ *h*(*K*))∥*x_s_*∥*RN_α_*∥*T_α_*)*GW* compares *M_Uβ_*_−_*_G_* with *M_Uβ_*_−__G_*. *GW* regards {*DID_β_*, *M_Uβ_*_−_*_G_*, *v_β_*, *T_α_*} as being sent from a legitimate user because *M_Uβ_*_−_*_G_* = *M_Uβ_*_−__G_*.


#### 3.2.2. Logging in with the Identity of a Legitimate User

We describe when *U_α_* impersonates a legitimate user *U_i_* who has registered with *GW* using *x_s_* and *h*(*K*).


Step 1In the previous session, when *U_i_* sends the authentication request {*DID_i_*, *M_Ui_*_−_*_G_*, *v_i_*, *T_i_*} to *GW* as shown in Section 2.2, *U_α_* eavesdrops on it.Step 2*U_α_* computes the following. *RN_α_* is a random nonce generated by *U_α_*. *T_α_* is the current timestamp of *U_α_* system. *x_s_* and *h*(*K*) are already known to *U_α_*, as mentioned above. *RN_i_*=*v_i_* ⊕ *x_s_* 
$h\left(ID_{i}\|H\_PW_{i}^{*}\|x_{s}\right)=DID_{i}⊕h\left(x_{s}\left|\left|RN_{i}\right|\right|T_{i}\right)$ 
$DID_{i}=h(ID_{i}\|H\_PW_{i}^{*}\|x_{s})⊕h(x_{s}\|RN_{i}\|T_{i})$ 
$A_{i}=h(ID_{i}\|H\_PW_{i}^{*}\|x_{s})⊕h(K)$ *M_Ui_*_−_*_G_* = *h*(*A_i_*∥*x_s_*∥*RN_α_*∥*T_α_*) *v_i_* = *RN_α_* ⊕ *x_s_**U_α_* sends the authentication request {*DID_i_*, *M_Ui_*_−_*_G_*, *v_i_*, *T_α_*} to *GW*.Step 3When *GW* receives {*DID_i_*, *M_Ui_*_−_*_G_*, *v_i_*, *T_α_*}, *GW* checks if (*T_G_*−*T_α_*) ≤ Δ*T*, where *T_G_* is the current timestamp of *GW* system. If (*T_G_*−*T_α_*) ≤ Δ*T*, then the next step proceeds; otherwise, this phase is aborted.Step 4*GW* computes the following. *RN_α_* = *v_i_* ⊕ *x_s_* X*=*DID_i_* ⊕ *h*(*x_s_*∥*RN_α_*∥*T_α_*) *M_Ui_*_−__G_* = *h*((X* ⊕ *h*(*K*))∥*x_s_*∥*RN_α_*∥*T_α_*)*GW* compares *M_Ui_*_−_*_G_* with *M_Ui_*_−__G_*. *GW* regards {*DID_i_*, *M_Ui_*_−_*_G_*, *v_i_*, *T_α_*} as being sent from a legitimate user because *M_Ui_*_−_*_G_*=*M_Ui_*_−__G_*.


### 3.3. Gateway Node Bypassing Attacks Using an Attacker's Own Smart Card

As discussed in Section 3.2, if an attacker *U_α_* obtains *x_s_* and *h*(*K*) using data stored in his/her own smart card, he/she can impersonate *GW*. The following shows the attack process in detail. *U_α_* denotes an attacker here.


Step 1Login phase begins when *U_i_* wants to access the WSN as described in Section 2.2.When *U_i_* sends the authentication request {*DID_i_*, *M_Ui_*_−_*_G_*, *v_i_*, *T_i_*} to *GW*, *U_α_* eavesdrops on the transmission.Step 2*U_α_* computes the following using *x_s_* and {*DID_i_*, *M_Ui_*_−_*_G_*, *v_i_*, *T_i_*}. *T_α_* and *T_α_′* denote the current timestamp of *T_α_* system, and *T_α_* < *T_α_′. U_α_* generates a random nonce *RN_α_*. S*ID_α_* is created by *U_α_*. *y_i_* = *RN_α_* ⊕ *x_s_* *M_G_*_−_*_Sj_* = *h*(*DID_i_*∥S*ID_α_*∥*x_s_*∥*T_α_*) 
$z_{i}^{*}=M_{G-S_{j}}⊕RN_{\alpha }$ 
$w_{i}=z_{i}^{*}⊕x_{s}$ *M_G_*_−_*_Ui_* = *h*(*DID_i_*∥*M_G_*_−_*_Sj_*∥*M_Ui_*_−_*_G_*∥*x_s_*∥*T_α_′*)*U_α_* forges the authentication request sent from *GW* to *U_i_* in authentication-key agreement phase using {*y_i_*, *w_i_*, *M_G_*_−_*_Ui_*, *T_α_′*}.Step 3When *U_i_* receives {*y_i_*, *w_i_*, *M_G_*_−_*_Ui_*, *T_α_′*} from *U_α_*, *U_i_* checks if (*T_U_′* − *T_α_′*) ≤ Δ*T*, where *T_U_′* is the current timestamp of *U_i_* system. If (*T_U_′* − *T_α_′*) ≤ Δ*T*, then the next step proceeds; otherwise, this phase is aborted.Step 4The smart card computes the following. *RN_α_* = *y_i_* ⊕ *x_s_* 
$z_{i}^{*}=w_{i}⊕x_{s}$ 
$M_{G-Sj}=z_{i}^{*}⊕RN_{\alpha }$ *M_G_*_−__Ui_*=h(*DID_i_*∥M*_G_*_−_*_Sj_*∥*M_Ui_*_−_*_G_*∥*x_s_*∥*T_α_′*)The smart card compares *M_G_*_−_*_Ui_*with *M_G_*_−__Ui_*. Since *M_G_*_−_*_Ui_*=*M_G_*_−__Ui_*, *U_i_* regards {*y_i_*, *w_i_*, *M_G_*_−_*_Ui_*,*T_α_′*} as being transmitted from *GW*. Therefore, *U_α_* can communicate with *U_i_* using the session key *K_s_* = *f*((*DID_i_*∥*RN_α_*), *x_s_*).


## 4. The Proposed Scheme

In this section, we propose an improved scheme that can overcome the security weaknesses presented in Section 3. The reason why Vaidya *et al.*'s scheme is vulnerable to sensor node capture attacks is that *x_s_* is stored in plaintext form in *S_j_* though it is a secret value. To make matters worse, *x_s_* is shared between all sensor nodes in the WSN. Also, in Vaidya *et al.*'s scheme, an attacker can compute and use *x_s_* and *h*(*K*) for attacks because they are stored in all users' smart cards. Therefore, the main ideas of our proposed scheme are as follows:

▪ When *GW* personalizes a smart card for *U_i_* in the registration phase, *GW* uses *Xs_i_* = *h*(*H*_*ID_i_* ∥*x_s_*) and *h*(*H*_*ID_i_*∥K; instead of *x_s_* and *h*(*K*) to prevent an attacker from computing *x_s_* or *h*(*K*). Since *Xs*_i_ and h *h*(*H*_*ID_i_*∥K; are unique for each user, an attacker cannot reuse them to impersonate a legitimate user.
▪ In the proposed scheme, $Xs_{j}^{*}=h(SID_{j}\|x_{s})$ instead of *x_s_* is stored in *S_j_*to prevent an attacker from extracting *x_s_* from *S_j_*. Since $Xs_{j}^{*}$ is unique for each sensor node, we can attenuate the effects of sensor node capture attacks as much as possible.

We describe each phase in detail in Sections 4.1 through 4.4. Before describing the proposed scheme in detail, we present the security requirements for the proposed scheme.

▪ The proposed scheme has to be secure against possible attacks such as replay, password guessing, user impersonation, gateway node bypassing and parallel session attacks.
▪ The proposed scheme has to minimize the damage caused by sensor node capture attacks. The authentication scheme cannot be a perfect solution that blocks sensor node capture attacks completely. Nevertheless, the proposed scheme should attenuate the effects of sensor node capture attacks as much as possible.
▪ We assume an attacker can obtain all data from a smart card. Therefore, our proposed scheme has to be devised considering stolen smart card attacks, lost smart card problems, and attacks that use an attacker's own smart card, as shown in Section 3.
▪ The proposed scheme must be secure against privileged-insider attacks or stolen-verifier attacks.
▪ The proposed scheme has to provide methods for mutual authentication, key agreement between *U_i_* and *S_j_*, and password change.

### 4.1. Registration Phase

**Figure 4.**
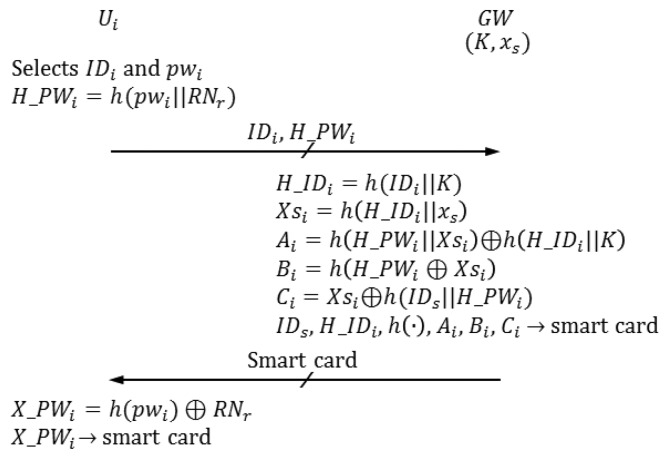
Registration phase of the proposed scheme.

In the registration phase, *U_i_* selects *ID_i_* and *pw_i_*. *U_i_* computes and sends the registration request {*ID_i_*, *h*(*pw_i_*)∥*RN_r_*)} to the gateway node, where *RN_r_* is a random nonce. Then, *GW* personalizes a smart card for *U_i_*. Figure 4 illustrates the registration phase of the proposed scheme. Meanwhile, *SID_j_* and $Xs_{j}^{*}$ are stored in *S_j_*, where $Xs_{j}^{*}=h(SID_{j}\|x_{s})$

before *S_j_* is deployed into a target field.


R-1*U_i_* selects *ID_i_* and *pw_i_*.R-2*U_i_* generates a random nonce *RN_r_* and computes *H*_*PW_i_* = *h*(*pw_i_*∥*RN_r_*).*U_i_* sends the registration request {*ID_i_*, *H*_*PW_i_*} to *GW* in secure channels.R-3*GW* computes the following when it receives a registration request from *U_i_*. *H*___*ID_i_* = *h*(*ID_i_*∥*K*) *Xs_i_* = *h*(*H*___*ID_i_*∥*x_s_*) *A_i_*=*h*(*H_PW_i_*∥*Xs_i_*) ⊕ *h*(*H*___*ID_i_* ∥*K*) *B_i_* = *h*(*H_PW_i_* ⊕ *Xs_i_*) *C_i_* = *X_Si_* ⊕ *h*(*ID_s_*∥*H_PW_i_*)*GW* personalizes the smart card with *ID_s_*, *H*___*ID_i_*, *h*(·),*A_i_,B_i_*and *C_i_*.*GW* sends the smart card to *U_i_* in secure channels.R-4*U_i_* computes *X*_*PW_i_* = *h*(*pw_i_*) ⊕ *RN_r_* and adds *X*_*PW_i_* to the smart card.


### 4.2. Login Phase

**Figure 5.**
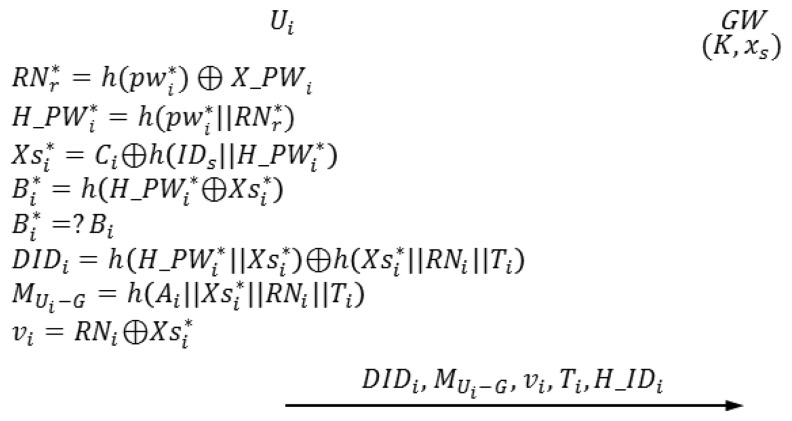
Login phase of the proposed scheme.

The login phase begins when *U_i_* inserts *U_i_*'s smart card into a terminal and inputs $ID_{i}^{*}$ and $pw_{i}^{*}$. In this phase, *U_i_* sends the authentication request to *GW*. Figure 5 depicts the login phase of the proposed scheme.


L-1*U_i_* inserts *U_i_*'s smart card into a terminal and inputs 
$ID_{i}^{*}$ and. 
$pw_{i}^{*}$L-2The smart card computes the following. 
$RN_{r}^{*}=h(pw_{i}^{*})⊕X\_PW_{i}$ 
$H\_PW_{i}^{*}=h(pw_{i}^{*}\|RN_{r}^{*})$ 
$Xs_{i}^{*}=C_{i}⊕h\left(ID_{s}\|H\_PW_{i}^{*}\right)$ 
$B_{i}^{*}=h\left(H\_PW_{i}^{*}⊕Xs_{i}^{*}\right)$The smart card compares 
$B_{i}^{*}$ with *B_i_*. If 
$B_{i}^{*}=B_{i}$, then the next step proceeds; otherwise, this phase is aborted.L-3The smart card generates a random nonce *R_Ni_* and computes the following. *T_i_* is the current timestamp of *U_i_* system. 
$DID_{i}=h\left(H\_PW_{i}^{*}\|Xs_{i}^{*}\right)⊕h\left(Xs_{i}^{*}\|RN_{i}\|T_{i}\right)$ 
$M_{U_{i}-G}=h\left(A_{i}\|Xs_{i}^{*}\|RN_{i}\|T_{i}\right)$ 
$v_{i}=RN_{i}⊕Xs_{i}^{*}$The smart card sends the authentication request {*DID_i_*, *M_Ui_*_−_*_G_*, *v_i_*, *T_i_*, *H*___*ID_i_* } to *GW*.


### 4.3. Authentication-Key Agreement Phase

**Figure 6.**
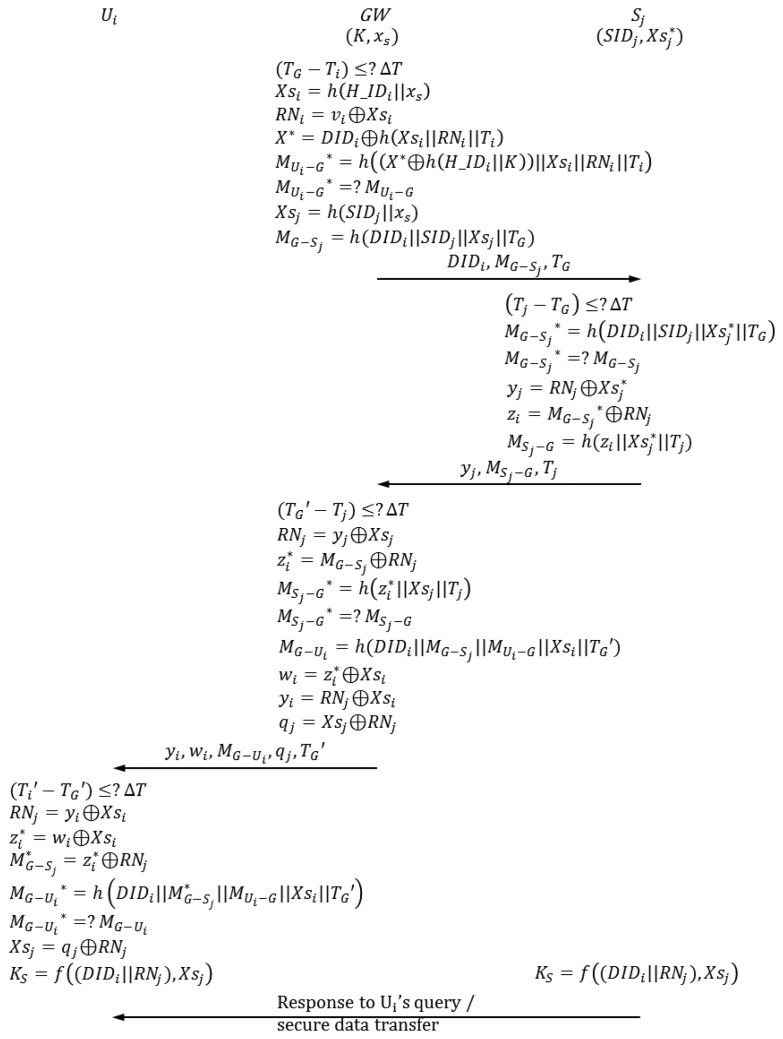
Authentication-key agreement phase of the proposed scheme.

When *GW* receives an authentication request from *U_i_*, the authentication-key agreement phase begins. In this phase, *U_i_*, *GW, S_j_* and send and receive authentication requests from one another. Figure 6 shows the authentication-key agreement phase of the proposed scheme. The following describes this process in detail.


A-1*GW* checks if (*T_G_* − *T_i_*) ≤ Δ*T*, where *T_G_* is the current timestamp of *GW* system.If (*T_G_* − *T_i_*) ≤ Δ*T*, then the next step proceeds; otherwise, this phase is aborted.A-2*GW* computes the following. *X_Si_* = *h*(*H*___*ID_i_*∥*x_s_*) *RN_i_* = *v_i_* ⊕ *Xs_i_* X* = *DID_i_* ⊕ *h*(*Xs_i_*∥*R_Ni_*∥*T_i_*) *M_Ui_*_−__G_* = *h*((X* ⊕ *h*(*H___ID_i_*∥*K*))∥*Xs_i_*∥*R_Ni_*∥*T_i_*)*GW* compares *M_Ui_*_−__G_* with *M_Ui_*_−_*_G_*. If *M_Ui_*_−__G_*=*M_Ui_*_−_*_G_*, then the next step proceeds; otherwise, this phase is aborted.A-3*GW* computes the following. *T_G_* is the current timestamp of *GW* system. *S_j_* is the nearest sensor node that can respond to *U_i_* 's request. *Xs_j_*=*h*(*SID_j_*∥*x_s_*) *M_G_*_−_*_Sj_* = *h*(*DID_i_*∥*SID_j_*∥*Xs_j_*∥*T_G_*)*GW* sends the authentication request {*DID_i_*, *M_G_*_−_*_Sj_*, *T_G_*} to S*_j_*.A-4*GW* checks if (*T _j_* − *T_G_*) ≤ Δ*T*, where *T_j_* is the current timestamp of *S_j_*.If (*T _j_* − *T_G_*) ≤ Δ*T*, then the next step proceeds; otherwise, this phase is aborted.A-5*S_j_* computes 
${M_{G-Sj}}^{*}=h\left(DID_{i}\left|\left|SID_{j}\right|\right|Xs_{i}^{*}\|T_{G}\right).$*S_j_* compares *M_G_*_−__Sj_* with *M_G_*_−_*_Sj_*. If *M_G_*_−__Sj_* = *M_G_*_−_*_Sj_*, then the next step proceeds; otherwise, this phase is aborted.A-6*S_j_* generates a random nonce *RN_j_* and computes the following. 
$y_{j}=RN_{j}⊕Xs_{j}^{*}$ *z_i_* = *M_G_*_−__Sj_*⊕*RN_j_* 
$M_{S_{j}-G}=h(z_{i}\|Xs_{j}^{*}\|T_{j})$*S_j_* sends the authentication request {*y_i_*, *M_Sj_*_−_*_G_*, *T_j_*} to *GW*.A-7*GW* checks if (*T_G_*′ − *T_j_*) ≤ Δ*T*, where *T_G_*′ is the current timestamp of *GW*.If (*T_G_*′ − *T_j_*) ≤ Δ*T*, then the next step proceeds; otherwise, this phase is abortedA-8*GW* computes the following. *RN_j_* = *y_j_* ⊕ *Xs_i_* 
$z_{i}^{*}=M_{G-S_{j}}⊕RN_{j}$ 
${M_{S_{j}-G}}^{*}=h\left(z_{i}^{*}\|Xs_{j}\|T_{j}\right)$*GW* compares *M_Sj_*_−__G_* with *M_Sj_*_−_*_G_*. If *M_Sj_*_−_*_G_* = *M_Sj_*_−_*_G_*, then the next step proceeds; otherwise, this phase is aborted.A-9*GW* computes the following: *M_G_*_−_*_Ui_* = *h*(*DID_i_* ∥*M_G_*_−_*_Sj_*∥*M_Ui_*_−_*_G_*∥*Xs_i_*∥*T_G_*′) 
$w_{i}=z_{i}^{*}⊕Xs_{i}$ *y_i_*=*RN_j_* ⊕ *Xs_i_* *q_j_*=*Xs_j_* ⊕ *RN_j_**GW* sends the authentication request {*y_i_*, *w_i_*, *M_G_*_−_*_Ui_*, *q_j_*, *T_G_*′)} to *U_i_*.A-10*U_i_* checks if (*T_i_*′−*T_G_*′) ≤ Δ*T*, where *T_i_*′ is the current timestamp of *U_i_*. If (*T_i_*′−*T_G_*′) ≤ Δ*T*, then the next step proceeds; otherwise, this phase is aborted.A-11The smart card computes the following: *RN_j_* =*y_i_* ⊕*Xs_i_* 
$z_{i}^{*}=w_{i}⊕Xs_{i}$ 
$M_{G-S_{j}}^{*}=z_{i}^{*}⊕RN_{j}$ 
${M_{G-U_{i}}}^{*}=h\left(DID_{i}\|M_{G-S_{j}}^{*}\|M_{U_{i}-G}\|Xs_{i}\|T_{G}\prime \right)$The smart card compares *M_G_*_−__Ui_* with *M_G_*_−_*_Ui_*. If *M_G_*_−__Ui_* =*M_G_*_−_*_Ui_*, then mutual authentication between *U_i_* and *SN_j_* is completed successfully; otherwise, this phase is aborted.A-12The smart card computes the following to get a session key for communication with *S_j_*. Meanwhile, *S_j_* also computes *K_S_* = *f*((*DID_i_* ∥*R_Ni_*), *Xs_j_* to share a session key with *U_i_*. *Xs_j_* = *q_j_* ⊕ *RN_j_* *K_s_* = *f*((*DID_i_*∥*RN_j_*), *Xs_j_*)


### 4.4. Password Change Phase

The password change phase proceeds when *U_i_* changes *U_i_* 's existing password to a new one. In the password change phase, *U_i_* does not have to communicate with *GW*.


P-1*U_i_* inserts its smart card into a terminal and inputs, 
$ID_{i}^{*}$, 
$pw_{i}^{*}$ and *pw_ni_*. is *U_i_*'s new password.P-2The smart card computes the following. 
$RN_{r}^{*}=h(pw_{i}^{*})⊕X\_PW_{i}$ 
$H\_PW_{i}^{*}=h(pw_{i}^{*}\|RN_{r}^{*})$ 
$Xs_{i}^{*}=C_{i}⊕h\left(ID_{s}\|H\_PW_{i}^{*}\right)$ 
$B_{i}^{*}=h\left(H\_PW_{i}^{*}⊕Xs_{i}^{*}\right)$The smart card compares 
$B_{i}^{*}$ with *B_i_*. If 
$B_{i}^{*}=B_{i}$, then the next step proceeds; otherwise, this phase is aborted.P-3The smart card computes the following. 
$H\_PW_{ni}=h(pw_{ni}\|RN_{r}^{*})$ 
$A_{ni}=A_{i}⊕h\left(H\_PW_{i}^{*}\|Xs_{i}^{*}\right)⊕h\left(H\_PW_{ni}\|Xs_{i}^{*}\right)$ 
$B_{ni}=h\left(H\_PW_{ni}⊕Xs_{i}^{*}\right)$ 
$C_{ni}=Xs_{i}^{*}⊕h(ID_{s}\|H\_PW_{ni})$The smart card replaces the existing values *A_i_*, *B_i_* and *C_i_* with the new values *A_ni_*, *B_ni_* and *C_ni_*.


## 5. Security Analysis of the Proposed Scheme

This section is devoted to the security analysis of our proposed scheme. We discuss the security of our proposed scheme in terms of the security requirements presented in Section 4. Table 2 shows a security comparison of the proposed scheme.

▪ **Replay attacks**: The proposed scheme resists replay attacks because all authentication requests include current timestamps, such as *T_i_* of {*DID_i_*, *M_Ui_*_−_*_G_*, *v_i_*, *T_i_*, *H_ID_i_*}.
▪ **User impersonation attacks and gateway node bypassing attacks**: In the proposed scheme, an attacker cannot create valid authentication requests {*DID_i_*, *M_Ui_*_−_*_G_*, *v_i_*, *T_i_*, *H*___*ID_i_*} and {*y_i_*, *w_i_*, *M_G_*_−_*_Ui_*,*q_j_*,*T_G_*′} because he/she cannot compute the secret data *x_s_* and *h*(*K*). Therefore, user impersonation attacks and gateway node bypassing attacks are impossible.
▪ **Parallel session attacks**: The proposed scheme is secure against parallel session attacks because all authentication requests include random nonces such as *DID_i_*, and *v_i_* of {*DID_i_*, *M_Ui_*_−_*_G_*, *v_i_*, *T_i_*, *H*___*ID_i_*}.
▪ **Password guessing attacks**: *pw_i_* cannot be guessed by an attacker because it is transmitted as the results which are concatenated with some secret values and one-way hashed. Even a privileged-insider cannot guess *U_i_*'s password from the registration request {*ID_i_*,*H*_*PW_i_*} because *RN_r_* in *H*_*PW_i_* = *h*(*pw_i_*∥*RN_r_*) is a unknown value to him/her.
▪ **Sensor node capture attacks**: Though an attacker captures a sensor node and obtains secret data *SID_j_* and $Xs_{j}^{*}$ from it, the attacker cannot impersonate *U_i_*, *GW*, or other sensor nodes. Since is the unique secret data only for *S_j_*, an attacker cannot compute *Xs_j_* for *U_i_* or *x_s_* for *GW*. In addition, he/she cannot compute the secret data of other sensor nodes except *S_j_*.
▪ **Stolen smart card attacks and lost smart card problems**: Though an attacker extracts *ID_s_*, *H*___*ID_i_*, h(·), *A_i_*, *B_i_*, *C_i_*, and *X*_*PW_i_* from *U_i_*'s smart card, he/she cannot compute any secret data *h*(*K*)or *x_s_* for attacks. Therefore, the proposed scheme is secure against stolen smart card attacks or lost smart card problems. In addition, though an attacker extracts, *ID_s_*, *H_i_D_α_*, *h*(·), *A_α_*, *B_α_*, *C_α_*, and *X*_*PW_α_* from his/her own smart card, he/she cannot compute any secret data *h*(*K*) or *x_s_* for attacks. Therefore, the proposed scheme prevents attacks using an attacker's own smart card.
▪ **Privileged-insider attacks**: The proposed scheme resists privileged-insider attacks because *pw_i_* is transmitted as a digest of some other secret components.
▪ **Stolen-verifier attacks**: The proposed scheme is secure against stolen-verifier attacks, since does not maintain a verifier table.
▪ **Mutual authentication, key agreement, and password change phase**: The proposed scheme provides mutual authentication, key agreement between *U_i_* and *S_j_*, and password change phase.

## 6. Performance Analysis of the Proposed Scheme

Table 3 shows the computation cost comparison of the proposed scheme. Das' scheme [3], Khan and Alghathbar's scheme [4], Vaidya *et al.*'s scheme [12], and the proposed scheme use only hash and XOR operations. We compare these schemes in terms of the number of hash and XOR operations. The proposed scheme needs seven hash operations more than Vaidya's *et al.*'s [12]. Nevertheless, one of our main concerns is the computation cost of a sensor node rather than that of the entire scheme, because sensor nodes are resource-constrained. The computation cost of in the proposed scheme is the same as that of Vaidya *et al.*'s [12]. This means that the computation cost increase of the entire scheme is negligible considering the enhanced security. Meanwhile, with respect to communication cost, the number of messages transmitted in the proposed scheme is four, which is the same as that of Vaidya *et al.*'s scheme.

## 7. Conclusions

We have proposed an improved mutual authentication and key agreement scheme to overcome the security weaknesses of Vaidya *et al.*'s scheme. The proposed scheme resists user impersonation attacks and gateway node bypassing attacks using secret data stored in an attacker's own smart card or a sensor. In addition, the proposed scheme prevents possible attacks such as replay attacks, parallel session attacks, password guessing attacks, sensor node capture attacks, stolen smart card attacks, lost smart card problems, privileged-insider attacks, and stolen-verifier attacks. The proposed scheme is also efficient in terms of computation and communication cost considering the limited resources of sensors.

## Figures and Tables

**Table 1.** Notations [12].

**Symbol**	**Description**
*U_i_*	*i*-th user
*S_j_*	*j*-th sensor node
*GW*	Gateway node
*ID_i_*	Identity of
*pW_i_*	Password of
*SID_j_*	Identity of
*ID_s_*	Identity of smart card
*K*	Secret key known to only
*X_s_*	Secret value generated by and shared between only and
*h*(·)	One-way hash function
*R_Ni_*	Random nonce of
*RN_j_*	Random nonce of
⊕	XOR operation
∥	Concatenation operation
=?, ≤?	Verification operation
*K_s_*	Session key
*f*(*x*,*k*)	Pseudo-random function of variable with key *k*
*T_i_*,*T_i_*′	Current timestamp of *U_i_*
*T_G_*,*T_G_*′	Current timestamp of *GW*
*T_j_*	Current timestamp of *S_j_*
Δ*T*	The maximum of transmission delay time permitted
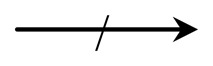	Secure channel
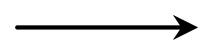	Insecure channel

**Table 2.** Security comparison of the proposed scheme.

**Security Features**	**Das' Scheme** [3]	**Khan and Alghathbar's Scheme** [4]	**Vaidya *et al.*'s Scheme** [12]	**The Proposed Scheme**
Replay attacks	Yes	Yes	Yes	Yes
User impersonation attacks	No	No	No	Yes
Gateway node bypassing attacks	No	No	No	Yes
Parallel session attacks	No	No	Yes	Yes
Password guessing attacks	No	No	Yes	Yes
Sensor node capture attacks	No	No	No	Yes
Stolen smart card attacks	No	No	Yes	Yes
Lost smart card problems	No	No	Yes	Yes
Privileged-insider attacks	No	Yes	Yes	Yes
Stolen-verifier attacks	Yes	Yes	Yes	Yes
Mutual authentication	No	No	Yes	Yes
Key agreement	No	No	Yes	Yes
Password change phase	No	Yes	Yes	Yes

(Yes: The scheme resists the attacks or provides the functionality; No: The scheme does not resist the attacks or provide the functionality).

**Table 3.** Computation cost comparison of the proposed scheme.

**Phases**		**Das' Scheme** [3]	**Khan and Alghathbar's Scheme** [4]	**Vaidya *et al.*'s Scheme** [12]	**The Proposed Scheme**
Registration phase	*U_i_*	0	1H	1H	2H + 1X
	*GW*	3H + 1X	2H + 1X	4H + 3X	6H + 3X
	*S_j_*	0	0	0	0

Login phase	*U_i_*	3H + 1X	3H + 1X	6H + 4X	7H + 5X
	*GW*	0	0	0	0
	*S_j_*	0	0	0	0

Authentication and key agreement phase	*U_i_*	0	0	1H + 3X	1H + 4X
	*GW*	4H + 2X	5H + 2X	6H + 6X	8H + 8X
	*S_j_*	1H	2H	2H + 2X	2H + 2X

Password change phase	*U_i_*	-	3H + 2X	8H + 6X	9H + 7X
	*GW*	-	0	0	0
	*S_j_*	-	0	0	0

Total		11H + 4X	16H + 6X	28H + 24X	35H + 30X

(H: The number of hash operations; X: The number of XOR operations).
